# Pattern and distribution of prenatally diagnosed congenital anomalies among high risk pregnant women in Ibadan, South Western Nigeria

**DOI:** 10.11604/pamj.2022.41.66.28874

**Published:** 2022-01-24

**Authors:** Janet Adetinuke Akinmoladun, Ibukun Deborah Famosaya, Godwin Inalegwu Ogbole

**Affiliations:** 1 Department of Radiology, College of Medicine, University of Ibadan, Ibadan, Oyo State, Nigeria,; 2 Department of Radiology, University College Hospital, Ibadan, Oyo State, Nigeria

**Keywords:** High risk, prenatal screening, pregnant women, ultrasound, congenital, anomalies

## Abstract

**Introduction:**

Congenital anomalies (CA) are structural or functional disorders present at birth. Routine prenatal ultrasound screening has become an indispensable tool for early detection of CA in developed countries which will facilitate appropriate preemptive actions for safe guarding the health of both mother and the unborn fetus. The prevalence of CA in the general population has been researched widely but very few studies exist on the prevalence of CA among high risk pregnancies. Aims and objective: the aim of this study was to determine the prevalence and pattern of congenital anomalies among high risk pregnant women in Ibadan, South West, Nigeria.

**Methods:**

this multicenter cross sectional study was conducted in three different hospitals in Ibadan, South West, Nigeria between August 2018 and July 2019. High risk pregnant women that met the inclusion criteria were recruited for the study. Participants underwent detailed fetal anomaly scans at gestational ages between 18-26 weeks during the study period using a Voluson P6 ultrasound machine (GE Healthcare Korea). Written informed consents were obtained from the participants. All the fetuses with ultrasound diagnosed congenital anomalies were followed up till either termination of pregnancy or delivery. Ethical approval was obtained for the study. The data were analyzed using SPSS.

**Results:**

a total of 418 high risk pregnant women underwent detailed fetal anomaly scan and CAs were detected in 13(3.1%) of them. Spontaneous abortion was the most common maternal risk factor reported although the association between it and congenital anomaly was not significant. The highest number of anomalies were detected in the genitourinary system while the least was in the central nervous system.

**Conclusion:**

the prevalence of high risk pregnant women is high in our environment but the incidence of CA among them is similar to that reported in the general population. Thus, prenatal ultrasonographic screening for congenital anomalies is necessary for all pregnant women irrespective of their risk status.

## Inroduction

Congenital anomaly (CA) is a broad term referring to any structural, functional or metabolic defect present at birth. The prevalence of congenital anomalies varies in different population of the world with a range of less than 1% up to 8% [1,2]. The cause of CA is multifactorial in 25% of fetuses affected, environmental pollution and exposure to ionizing radiation are also implicated while the cause is still unknown in 60%4 of cases [3-5]. The introduction of ultrasound for prenatal detection of CA over 5 decades ago, has seen remarkable advancement in technology and now remains an invaluable screening and diagnostic tool for congenital disorders [6,7].

Some medical and physical conditions present before or develop during pregnancy which predispose the fetus or mother to complications. Such pregnancies are considered to be high risk pregnancies and they include hypertension in pregnancy, gestational diabetes, seizure disorders, recurrent spontaneous abortions, advanced maternal age and teenage pregnancy [8,9]. A lot of research work has been done on the prevalence of CA in the general population of pregnant women, [10-13] however, limited information exists on the prevalence of these anomalies among the high risk pregnant women. The aim of this study is to determine the prevalence of CA among high risk pregnant women in Ibadan and to determine the pattern of anomalies common among them.

## Methods

This multicenter prospective study was conducted in Ibadan, South Western region of Nigeria between August 2019 and July 2020. Three major hospitals which are the main referral centers serving the city of Ibadan were selected for this study and patients were recruited from the antenatal clinics. These are University College Hospital (UCH), Adeoyo Maternity Teaching Hospital (AMTH) and Our Lady´s Apostle Catholic Hospital (OLACH). There was no documented report on the prevalence of congenital abnormalities among high risk women in Nigeria or Africa. The sample size was calculated with an assumption of 50% prevalence which yielded 380, and allowing for 10% attrition rate, a total of 418 high risk women were recruited for the study.

High risk pregnant women with gestational age between 18 and 26 weeks were recruited for the study. A pregnant woman was considered to be high risk if she was less than 20 years old or more than 34 years old, has had spontaneous abortion or birth defect in previous pregnancies, previous history of intrauterine fetal death, history of medical conditions like hypertension, diabetic mellitus, mental disorder, HIV infection or hepatitis in the index pregnancy. Written informed consents were taken from all the women that met the inclusion criteria and they were assured of privacy and confidentiality of their data. A detailed mid trimester trans-abdominal obstetric ultrasound scan was then performed using a Voluson P6® ultrasound machine (GE Healthcare, Korea). The guidelines of the International Society for Ultrasound in Obstetrics and Gynecology (ISUOG) [14] for performing mid-trimester ultrasound scan was used.

Congenital anomalies were defined as any structural abnormalities that occur during intrauterine life which were identified prenatally using ultrasonography. Once an anomaly was detected, it was classified into either mild, severe or lethal anomaly. Mild anomaly is one that requires medical/surgical intervention after delivery but life expectancy is good. Severe anomaly is one that causes handicap or death without medical/surgical intervention while a lethal anomaly is one that causes stillbirth or infant death with/without intervention. The managing obstetrician was informed and the nature of the defect was explained to the pregnant woman and her husband (if present). The woman is then counselled and left to make an informed decision on the pregnancy. The pregnancies were followed up depending on the decision of the woman, either till delivery or pregnancy termination, in case of severe anomalies.

Patients´ demographics, risk factors and detected anomalies were recorded in a data sheet. All recorded images of anomalies generated from the research were archived on a CD-ROM with back- ups on a personal computer hard drive and an external hard drive for possible future references. The data were analyzed using statistical package for social sciences (SPSS) software version 21 (SPSS Inc. Chicago, II USA.). Standardized formats like frequency tables, percentages and graphs were used to describe the prevalence of CA among high risk women and the distribution among the different body systems. The relationship between CA and maternal risk factors were tested using Chi-square and a P-value of < 0.05 was considered significant. Ethical approvals were obtained from University of Ibadan/University College Hospital Research Ethics Committee and Oyo State Research ethics committee and the reference number was UI/EC/17/0073.

## Results

Four hundred and eighteen (418) high risk pregnant women presented for fetal anomaly scan during the study period. Their ages ranged between 18 and 55 years with mean and standard deviation of 32.97±5.7 years. Equal number of participants (205, 49%) were noted in the 20-34 and 35-55 age groups, while lowest number (8,1.9%) of participants were teenagers. The gestational age(GA) at presentation of most of the women (318,76.1%) were between 18 and 24 weeks GA (Table 1). Thirteen CAs were detected among the 418 high risk pregnant women who presented for fetal anomaly scan accounting for 3.1% among the scanned population. Regarding the distribution of congenital anomalies among the high risk pregnancies, 4(30.76%) of the fetuses with anomalies were in women with Spontaneous abortion in previous pregnancies, two (15.38%) of them were in women with advanced age (>35 years). Three (23.08%) of the women with fetuses with CAs had multiple risk factors while one (7.69%) fetus each with CA was found in women with a child with birth defect, diabetes mellitus, psychiatry disorder and teenage pregnancy (Table 2). There was however no statistically significant association between maternal risk factors and congenital anomalies (t= 4.069, p=0.1).

**Table 1:** the socio-demographic characteristics of the high risk pregnant women that underwent prenatal screening for fetal anomaly scans (N=418)

Variable	Number	Percentage (%)
**Age (years)**		
15-19	8	2.00
20-34	205	49.00
35-55	205	49.00
Mean/SD	32.97±5.7	
**Education**		
No formal	1	0.20
Primary	4	1.00
Secondary	73	19.40
Tertiary	304	72.73
Unknown	36	8.6
**Total**	418	100
**Gestational age (weeks)**		
18-24	318	76.1
Above 25	100	23.9
Total	418	100

**Table 2:** the frequency of the detected congenital anomalies among the fetuses and the associated maternal risk factors

Risk factor	Frequency	Percentage (%)
Teenage pregnancy	1	7.69
Advanced maternal age	2	15.38
Previous spontaneous abortions	4	30.76
Previous child with birth defect	1	7.69
Diabetic mellitus	1	7.69
Psychiatry disorder	1	7.69
Multiple risk factors	3	23.08
Total	13	100

**Figure 1:**
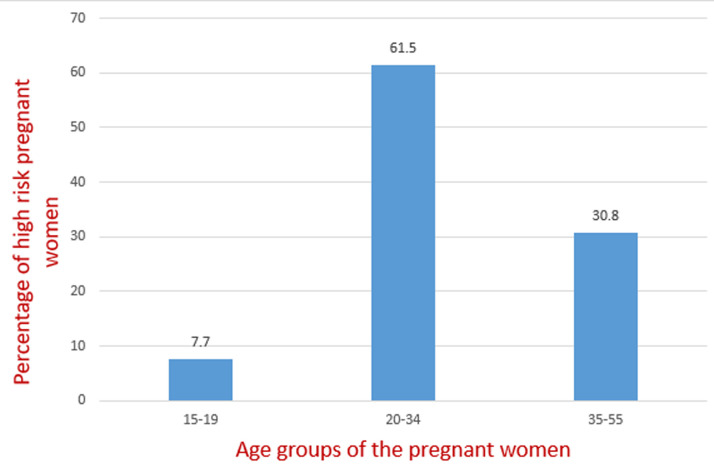
the percentage of high risk pregnant women with congenital anomalies in the different age groups

Eight (61.5%) fetuses with congenital anomalies were detected in the 20-34 age group while 4 fetuses had congenital anomalies in those above 34 representing 30.8% of the total CAs. 1(7.7%) of the teenagers had a fetus with CA (Figure 1). Abnormalities of the genitourinary system occur most frequently accounting for 4(30.77%) of the all the congenital anomalies. Cardiovascular anomalies were detected in 3(23.08%), while Two (15.41%) were detected in the musculoskeletal system of the fetuses. One (7.69%) anomaly each was noted in the central nervous system, respiratory system and gastrointestinal system. One (7.6%) fetus had anomalies involving multiple systems (Table 3).

**Table 3:** the distribution of the specific anomalies detected according to the affected systems, the maternal risk factors, severity of the anomalies and postnatal confirmation

System affected	Frequency (%)	Risk factor	Severity	Confirmed
**Central nervous system**	1 (7.69)			
Exencephaly	1 (100.0)	Previous birth defect	Lethal	Yes
**Cardiovascular system**	**3 (23.08)**			
Pericardial effusion/cardiomegaly	1 (33.33)	Teenage pregnancy	Severe	No
Hypo plastic left heart syndrome	1 (33.33)	Multiple risk factors	Severe	No
Atrioventricular septal defect	1 (33.33)	Diabetes mellitus	Severe	No
**Gastrointestinal system**	**1 (7.69)**			
Major Omphalocele	1 (100.0)	Multiple risk factors	Severe	Yes
**Genitourinary system**	**4 (30.77)**			
1.Bilateral severe hydronephrosis	1(25.0)	Previous spontaneous abortion	Severe	Yes
2.Bilateral moderate hydronephrosis	1 (25.0)	Advanced maternal age	Severe	Yes
3.Multicystic dysplastic kidneys disease	1 (25.0)	Psychiatry disorder	Severe	No
4. Megacystis	1 (25.0)	Previous spontaneous abortion	Severe	No
**Musculoskeletal system**	**2 (15.38)**			
1.Thanatophoric dysplasia	1 (50.0)	Advanced maternal age	Lethal	Yes
2. Achondroplasia	1 (50.0)	Previous spontaneous abortion	Severe	Yes
**Respiratory system**	**1 (7.7)**			
Sequestrated lung	1 (100)	Multiple risk factors	Mild	No
**Multiple anomalies**	**1 (7.7)**			
Limb-body wall defect	1 (100)	Previous spontaneous abortion	Lethal	Yes
**Total**	**13 (100)**			

**Figure 2:**
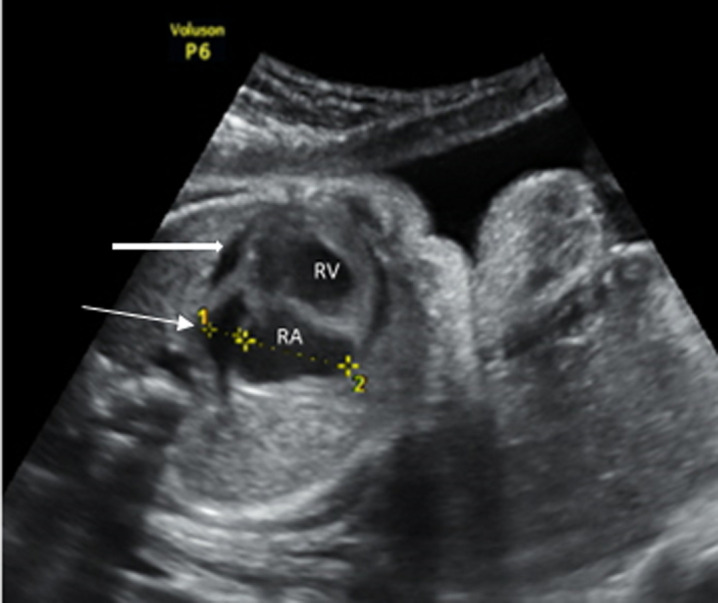
four chamber view of the heart of a fetus at 24-week GA; the block and slim arrows show hypoplastic left ventricle and left atrium respectively; the findings are consistent with hypoplastic left heart syndrome, a lethal congenital anomaly (RV= right ventricle, RA= right atrium)

**Figure 3:**
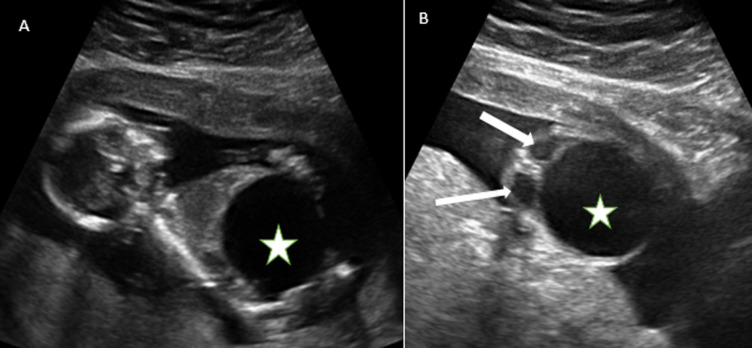
sagittal (A) and transverse (B) ultrasound scan images of a fetus at 18 week gestational age showing gross distended urinary bladder (star) consistent with megacystis - a severe fetal anomaly and dilated renal pelves bilaterally (arrows)

**Figure 4:**
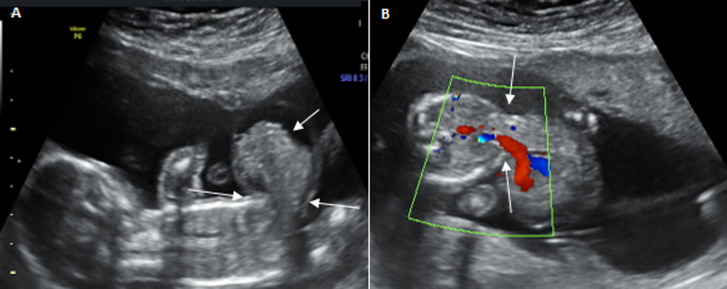
prenatal ultrasound scan images of a fetus at 20-week-GA with omphalocele, a severe congenital anomaly, in a 44-year-old woman; sagittal (A) and transverse (B) views of the fetus showing a defect in the anterior abdominal wall (arrows) with extrusion of the abdominal content through the defect and a membrane is seen to cover the abdominal content; the umbilical vein is seen centrally within the extruded abdominal contents (red colour)

The only major anomaly detected in the central nervous system was exencephaly while anomalies of the cardiovascular system include hypoplastic left heart syndrome (Figure 2), atrioventricular septal defect and pericardial effusion with cardiomegaly. Pulmonary sequestration, right polycystic kidney and megacystis with hydronephrosis (Figure 3) were the structural anomalies of the genitourinary detected during the study. Anomalies involving the musculoskeletal system were thanatophoric dysplasia and achondroplasia. Omphalocele (Figure 4) was the only anomaly seen in the gastrointestinal system while one of fetuses had limb body wall complex, which involved multiple systems. Nine (69.23%) of the CAs detected were severe, 1 (7.7%) was mild while 3(23.08%) were lethal (Table 3). Six (46.15%) of these anomalies were confirmed after delivery or termination of pregnancy (Table 3). These include exencephaly, omphalocoele, thanathophoric dysplasia and limb body wall defect. The lethal anomalies were detected in mothers with previous birth defects, advanced maternal age and previous spontaneous abortions. The only mild anomaly was found in a woman with multiple risk factors (Table 4).

**Table 4:** the relationship between the severity of the detected congenital anomalies and the identified risk factors

Risk	Pattern			Total (%)
	Mild (%)	Severe (%)	Lethal (%)	
Teenage pregnancy	0 (0)	1 (100.0)	0 (0)	1 (7.7)
Advanced maternal age	0 (0)	1 (50.00)	1 (50.00)	2 (15.4)
Previous spontaneous abortion	0 (0)	3 (23.1)	1 (7.7)	4 (30.8)
Previous child with defect	0 (100.0)	0 (0)	1 (0)	1 (7.7)
Diabetes mellitus	0 (0)	1 (7.7)	0 (0)	1 (7.7)
Psychiatry disorder	0 (0)	1 (7.7)	0 (0)	1 (7.7)
Multiple risk factors	1 (0)	2 (33.3)	0 (66.7)	3 (23.1)
**Total**	1 (7.7)	9 (53.8)	3 (38.4)	13 (100)

## Discussion

The prevalence of congenital anomalies varies in the general population of the world with a range of less than 1% up to 8% [1,2]. Many of the studies available determined the prevalence of CA among the general population after which the risk factors for the CAs were analyzed. Only very few studies are available on the prevalence of congenital anomalies among high risk pregnant women. The only study we could lay our hands upon was by Sunitha *et al*. [15] which was done in a large population in India, they got a prevalence of 11% among high risk pregnant women which is higher than the prevalence among the general population. The prevalence of congenital anomalies from this study was 3.1%, which falls within the general prevalence of the world. It was however significantly lower than what was recorded by Sunitha *et al*. [15]. The higher prevalence in India was attributed to parental consanguinity, which is a major contributor to congenital anomalies in that region, a factor that is nearly non-existence in the South Western region of Nigeria where our study was carried out. Similarly, the larger population of the study in India over a longer study period might have been more favorable for detection of congenital anomalies in their study.

Even though the causes of CAs are not known in more than 60% of cases, some maternal risk factors have been associated with increase occurrence. These include medical conditions in pregnancy e.g. hypertension in pregnancy, gestational diabetes and seizure disorders. Other risk factors are recurrent spontaneous abortions, previous baby with birth defect, consanguinity, advanced maternal age and teenage pregnancy. The causes are however multifactorial in about 25% of cases [3-5]. The commonest risk in our study was recurrent spontaneous abortion which was identified in about 30% of the fetuses with CAs. This was followed closely by the women with multiple risk factors recorded in 23% of the fetuses with anomalies.

Some studies have shown the association between advanced maternal age and chromosomal abnormalities like Down syndrome [5,6]. However, there are doubts whether there is a relationship between advanced maternal age and congenital structural anomalies. In this study, majority of the fetuses with anomalies (66.1%) were found in mothers between 20-34 years of age. Only 23.1% of fetuses with congenital anomalies were detected in the mothers ≥35 years. These findings are comparable to a survey done in Northern Nigeria by Singh and colleagues [16] where 20.8% of the congenital anomalies were reported in mothers with advanced maternal age (≥35years) while 66.6% were noted in mothers between 20-34 years. In the United States of America, Goetzinger *et al*. [17] also reported a decrease in occurrence of structural anomalies with increasing maternal age and they proposed an “all or nothing” phenomenon as a possible explanation for their findings. This suggests that early exposure of embryo to insult before organogenesis will either lead to embryonic death or normal fetal development. Other explanations given include early antenatal care, reduced substance use and increased prenatal vitamin use which are more likely to be present in pregnant mothers with advanced maternal age.

Teenage pregnancy is one of the high risk pregnancies because of anemia and nutritional deficiency that the teenagers are prone to [18]. In this study, only one of the thirteen congenital anomalies was identified in a teenage pregnant mother and this anomaly was in the cardiovascular system. In contrast, a prospective review by Singh *et al*. [16] in Northern Nigeria where a total of 10,163 neonates delivered in a tertiary hospital over a period of 3 years were examined for congenital anomalies. Teenage pregnancy accounted for 12.5% of the 72 congenital anomalies detected in their study, of which the central nervous system abnormality was the most prevalent. This disparity could be attributed to variation in sample size and the methods of both studies.

The risk of congenital anomalies is increased with a positive family history; this risk is even stronger when siblings are involved or if the woman herself has had at least a child with congenital anomalies [19]. Two of the mothers with fetuses with congenital anomalies previously had at least a child with congenital anomaly, although one of them had other risk factors. Sozan *et al*. [19] in a study from Egypt observed a significant association between birth defect and family history and they emphasized the role of genetics in the aetiology of congenital anomalies. This study however, did not show family history of congenital anomalies in previous pregnancy to be statistically significant.

Some maternal medical conditions have been associated with increased risk of having a baby with anomaly [4,20,21]. In this study, two women, each with diabetes mellitus and psychiatry disorder, had fetuses with CAs. The mechanism of teratogenicity in diabetes is multifactorial, hyperglycaemia and vascular complications which may lead to hypoxia and impaired clearance of toxins have been suggested [4]. Studies by Ozumba *et al*. [20] in Nigeria and Mill *et al*. [4] in USA reported higher incidence of congenital anomalies in mothers with diabetic mellitus. The study by John *et al*. [21] in Port Harcourt, Nigeria, was however at variance with this observation because none of the 456 diabetic mothers studied had babies with CAs. The reason for this could not be ascertained. Administration of antipsychotic drugs during pregnancy and substance abuse have been identified as causes of CAs in psychiatry patients. Pereira *et al*. [22] in a metanalysis involving a total of 4,194 children of mothers with mental illness and 249,548 children of mothers with no such disorders, concluded that there was a significant association between exposure to mental illness in mothers and risk of malformations in newborns.

Abnormalities of the genitourinary system were the commonest CAs in this study. This is similar to findings from an earlier work done in Nigeria by Akinmoladun *et al*. [23] and in Northern Nigeria by Anyanwu *et al*. [24]. However, Ekanem *et al*. [2] reported central nervous and skeletal system anomalies as the predominant systems affected by congenital anomalies in their review and this was attributed to possible environmental pollution form recurrent crude oil spillage in the part of the country. Similarly, Mashuda *et al*. [3] carried out a survey on the pattern of congenital anomalies in new-borns in Tanzania and reported the preponderance of central nervous system anomalies but this was attributed to inadequate use of folic acid by mothers of affected neonates. Prenatal ultrasonographic screening for congenital anomalies aids early detection of structurally malformed fetuses thus giving the parents ample time to reflect and plan for the unborn child. It also helps in making informed decision on prenatal therapeutic interventions where it is indicated. Furthermore, following extensive research and documentation of epidemiology of prenatal congenital anomalies in our environment, development of policies that will improve the antenatal care services in our society can be introduced.

**Limitations:** some of the anomalies were not confirmed, especially those that were internal, because post mortem autopsy is not routinely done for abortus in our environment and women with stillbirths most of the time do not agree to do autopsy.

## Conclusion

The prevalence of congenital anomalies in this study is similar to that reported in the general population of pregnant women. Therefore, prenatal ultrasonographic screening for congenital anomalies is necessary for all pregnant women irrespective of their risk status.

### What is known about this topic

- *The prevalence of congenital anomaly in the general population ranges between 1% and 8%;*
- *Some maternal risk factors like advanced maternal age, medical conditions and history of child with anomalies, increase the risk of occurrence of fetal anomalies*.

### What this study adds

- *The prevalence of anomaly among high risk women in this study is similar to the prevalence in the general population;*
- *Prenatal screening for fetal anomalies should be offered every pregnant woman irrespective of the presence of a risk factor since the incidence of anomalies among them is similar to that in the general population*.
